# The Killing Mechanism of Teixobactin against Methicillin-Resistant Staphylococcus aureus: an Untargeted Metabolomics Study

**DOI:** 10.1128/mSystems.00077-20

**Published:** 2020-05-26

**Authors:** Maytham Hussein, John A. Karas, Elena K. Schneider-Futschik, Fan Chen, James Swarbrick, Olivia K. A. Paulin, Daniel Hoyer, Mark Baker, Yan Zhu, Jian Li, Tony Velkov

**Affiliations:** a Department of Pharmacology and Therapeutics, School of Biomedical Sciences, Faculty of Medicine, Dentistry and Health Sciences, The University of Melbourne, Parkville, VIC, Australia; b The Florey Institute of Neuroscience and Mental Health, The University of Melbourne, Parkville, VIC, Australia; c Department of Molecular Medicine, The Scripps Research Institute, La Jolla, California, USA; d Discipline of Biological Sciences, Priority Research Centre in Reproductive Biology, Faculty of Science and IT, University of Newcastle, Callaghan, NSW, Australia; e Monash Biomedicine Discovery Institute, Department of Microbiology, Monash University, Clayton, VIC, Australia; University of Tennessee at Knoxville

**Keywords:** antimicrobial resistance, antimicrobial peptides, solid-phase peptide synthesis, teixobactin, metabolomics, methicillin-resistant *Staphylococcus aureus*

## Abstract

Antibiotics have served humankind through their use in modern medicine as effective treatments for otherwise fatal bacterial infections. Teixobactin is a first member of newly discovered natural antibiotics that was recently identified from a hitherto-unculturable soil bacterium, Eleftheria terrae, and recognized as a potent antibacterial agent against various Gram-positive bacteria, including methicillin-resistant Staphylococcus aureus (MRSA) and vancomycin-resistant enterococci. The most distinctive characteristic of teixobactin as an effective antibiotic is that teixobactin resistance could not be evolved in a laboratory setting. It is purported that teixobactin’s “resistance-resistant” mechanism of action includes binding to the essential bacterial cell wall synthesis building blocks lipid II and lipid III. In the present study, metabolomics was used to investigate the potential metabolic pathways involved in the mechanisms of antibacterial activity of the synthetic teixobactin analogue Leu_10_-teixobactin against a MRSA strain, S. aureus ATCC 700699. The metabolomes of S. aureus ATCC 700699 cells 1, 3, and 6 h following treatment with Leu_10_-teixobactin (0.5 μg/ml, i.e., 0.5× MIC) were compared to those of the untreated controls. Leu_10_-teixobactin significantly perturbed bacterial membrane lipids (glycerophospholipids and fatty acids), peptidoglycan (lipid I and II) metabolism, and cell wall teichoic acid (lipid III) biosynthesis as early as after 1 h of treatment, reflecting an initial activity on the cell envelope. Concordant with its time-dependent antibacterial killing action, Leu_10_-teixobactin caused more perturbations in the levels of key intermediates in pathways of amino-sugar and nucleotide-sugar metabolism and their downstream peptidoglycan and teichoic acid biosynthesis at 3 and 6 h. Significant perturbations in arginine metabolism and the interrelated tricarboxylic acid cycle, histidine metabolism, pantothenate, and coenzyme A biosynthesis were also observed at 3 and 6 h. To conclude, this is the first study to provide novel metabolomics mechanistic information, which lends support to the development of teixobactin as an antibacterial drug for the treatment of multidrug-resistant Gram-positive infections.

**IMPORTANCE** Antimicrobial resistance is one of the greatest threats to the global health system. It is imperative that new anti-infective therapeutics be developed against problematic “superbugs.” The cyclic depsipeptide teixobactin holds much promise as a new class of antibiotics for highly resistant Gram-positive pathogens (e.g., methicillin-resistant Staphylococcus aureus [MRSA]). Understanding its molecular mechanism(s) of action could lead to the design of new compounds with a broader activity spectrum. Here, we describe the first metabolomics study to investigate the killing mechanism(s) of teixobactin against MRSA. Our findings revealed that teixobactin significantly disorganized the bacterial cell envelope, as reflected by a profound perturbation in the bacterial membrane lipids and cell wall biosynthesis (peptidoglycan and teichoic acid). Importantly, teixobactin significantly suppressed the main intermediate d-alanyl-d-lactate involved in the mechanism of vancomycin resistance in S. aureus. These novel results help explain the unique mechanism of action of teixobactin and its lack of cross-resistance with vancomycin.

## Abstract (precis)

Antimicrobial resistance is one of the greatest threats to the global health system. It is imperative that new anti-infective therapeutics be developed against problematic “superbugs.” The cyclic depsipeptide teixobactin holds much promise as a new class of antibiotics for highly resistant Gram-positive pathogens (e.g., methicillin-resistant Staphylococcus aureus [MRSA]). Understanding its molecular mechanism(s) of action could lead to the design of new compounds with a broader activity spectrum. Here, we describe the first metabolomics study to investigate the killing mechanism(s) of teixobactin against MRSA. Our findings revealed that teixobactin significantly disorganized the bacterial cell envelope, as reflected by a profound perturbation in the bacterial membrane lipids and cell wall biosynthesis (peptidoglycan and teichoic acid). Importantly, teixobactin significantly suppressed the main intermediate d-alanyl-d-lactate involved in the mechanism of vancomycin resistance in S. aureus. These novel results help explain the unique mechanism of action of teixobactin and its lack of cross-resistance with vancomycin.

## INTRODUCTION

Antimicrobial resistance has emerged as one of the major global health problems that will inevitably lead to rising economic and social costs in the billions of dollars. The World Health Organization (WHO) has warned of the possibility of a postantibiotic era where the achievements of modern medicine are undermined by common bacterial infections that have become untreatable (1). The Infectious Diseases Society of America (IDSA) has listed Staphylococcus aureus isolates (methicillin resistant, vancomycin intermediate, and vancomycin resistant) as being among the top six most dangerous multidrug-resistant (MDR) microorganisms, requiring the urgent development of antibiotics (2). Resistance of these Gram-positive “superbugs” to last-resort antibiotics such as vancomycin has been increasingly reported in community and hospital settings (3). Hence, there is an urgent need for the development of antibiotics with efficacy against resistant pathogens and with novel mechanisms of action that act on targets that are more resistant to mutation, i.e., “resistant to resistance” (4).

**FIG 1:**
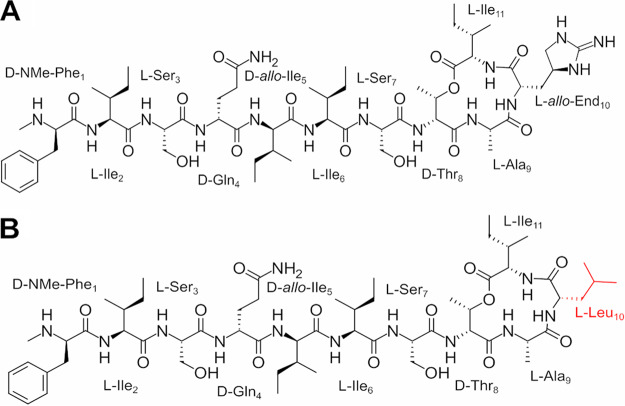
Chemical structures of native teixobactin (A) and Leu_10_-teixobactin (B).

Nature has created a wonderful serendipity for humankind in teixobactin, an antimicrobial peptide isolated from a previously uncultivable species of soil bacterium named Eleftheria terrae (5). Teixobactin is a macrocyclic depsipeptide consisting of 11 amino acids and possesses a 13-membered macrocycle between the side chain of d-threonine 8 (d-Thr8) and the C terminus (Fig. 1). The structure contains five nonproteinogenic amino acids, including the rare l-*allo*-enduracididine (l-*allo-*End) and four d-amino acids, *N*-methyl-d-phenylalanine 1 (*N*-Me-d-Phe_1_), d-glutamine 4 (d-Gln_4_), d-*allo*-isoleucine 5 (d-*allo*-Ile_5_), and d-Thr_8_ (6–10). Native teixobactin possesses narrow-spectrum bactericidal activity against several Gram-positive pathogens such as S. aureus and Mycobacterium tuberculosis (5). Teixobactin exhibits no cross-resistance with vancomycin while also displaying superior killing against late-exponential-phase bacterial cells (5, 11).

Given that the peptidoglycan component of the bacterial cell envelope is crucial for maintaining structural integrity, it is a key target for a number of antibiotics, including β-lactams and vancomycin (12). The Gram-positive bacterial cell wall is composed of peptidoglycan and cell wall teichoic acids (13). Peptidoglycan consists of linear chains of two alternating amino-sugars, namely, *N-*acetylglucosamine (GlcNAc) and *N*-acetylmuramic acid (MurNAc), which are covalently linked and decorated with a pentapeptide chain. A cross-linked matrix of such chains constitutes the cell wall backbone; this matrix structure confers elasticity that allows the cell to resist osmotic pressure (14). Peptidoglycan synthesis starts with the formation of uridine diphosphate-*N*-acetylmuramate (UDP-MurNAc) functionalized within the pentapeptide segment; this is followed by coupling to an undecaprenyl pyrophosphate moiety to form lipid I. Uridine diphosphate *N*-acetylglucosamine (UDP-GlcNAc) is then coupled to lipid I to form lipid II; this assembly takes place on the cytosolic side of the bacterial membrane. Subsequently, lipid II is then translocated across the periplasm, wherein penicillin-binding proteins catalyze transglycosylation and transpeptidation to merge the peptidoglycan subunits into the cell wall. Undecaprenyl pyrophosphate is then shuttled back to the cytosol, where it is dephosphorylated and recycled into another round of elongation (15, 16). Lipid II is susceptible to attack from antimicrobial agents while it is exposed on the outside of the bacterial inner membrane. Its structure presents multiple binding sites for antibiotic agents, such as the C terminus of its pentapeptide domain, which is targeted by vancomycin (17), or the pyrophosphate moiety, which is targeted by lantibiotics and ramoplanin (18). Only a small quantity of lipid II exists in the cytoplasmic membrane at any given time (12), which is reflective of a high turnover rate and/or rapid utilization for cell wall formation. As the rate-limiting step in peptidoglycan biogenesis, inhibition of lipid II effectively cripples cell wall synthesis and repair, thus leading to cell death via autolysis (15). Notably, the lipid II structure is unique within the domain *Bacteria*, thereby conferring high target specificity for lipid II binders and potentially avoiding mammalian cell toxicity (16). Teixobactin possesses a unique mechanism of action whereby it disrupts bacterial cell wall synthesis through binding and inhibition of the lipid II and lipid III substrates, which are critical for the biosynthesis of the peptidoglycan (5, 19). Gram-negative bacteria are surrounded by a thin layer of peptidoglycan (∼1 to 3 layers), whereas the peptidoglycan of Gram-positive bacteria is extremely thick (∼20 layers). In contrast, the outer membrane of Gram-negative bacteria is decorated with lipopolysaccharide (LPS), making it highly impermeable to noxious substances. This presents a possibility that teixobactin is inactive against Gram-negative bacteria as it cannot permeate the robust outer membrane. Neither plating of S. aureus on media with subinhibitory levels of teixobactin nor serial passaging over 27 days resulted in resistant mutants (5). Teixobactin appears to bind to the pyrophosphate moiety of lipid II but not to mature peptidoglycan, which lacks pyrophosphate (15); this could account for the lack of cross-resistance with vancomycin (5).

While the total synthesis of native teixobactin has been achieved, its preparation is difficult due to the need for the appropriate l-*allo*-End precursor, which is synthetically challenging to obtain in useful quantities (7, 20, 21). Leu_10_-teixobactin has emerged as an analogue that has activity comparable to that of native teixobactin and is generally accepted as a suitable substitute by researchers in the field (22–24). The present report presents the first study aimed at elucidating the bacterial killing mechanism(s) of the Leu_10_-teixobactin analogue against the methicillin-resistant S. aureus (MRSA) strain ATCC 700699 using untargeted metabolomics studies.

## RESULTS AND DISCUSSION

### MIC of Leu_10_-teixobactin.

Leu_10_-teixobactin was screened for direct antimicrobial activity against a panel of methicillin-resistant and vancomycin-intermediate S. aureus (VISA) strains. Leu_10_-teixobactin displayed effective bactericidal activity against all strains, with little difference between the susceptible S. aureus strain ATCC 29213 (MIC, 1 μg/ml) and methicillin-resistant S. aureus ATCC 700698 and ATCC 700699 (MIC, 1 μg/ml). Leu_10_-teixobactin also showed the same activity against VISA JKD6008 (MIC, 1 μg/ml) and slightly increased activity against VISA JKD6009 (MIC, 0.5 μg/ml).

### Antibacterial killing kinetics of Leu_10_-teixobactin.

The killing kinetics of Leu_10_-teixobactin in static time-kill studies was assessed against methicillin-resistant S. aureus ATCC 700699 (MIC, 1 μg/ml) by using different concentrations (0.25× MIC, 0.5× MIC, 1.0× MIC, 2.0× MIC, and 4× MIC) across multiple time points (0.5, 1, 2, 4, 8, and 24 h) (see Fig. S1A in the supplemental material). The 0.25-μg/ml (0.25× MIC) concentration of Leu_10_-teixobactin displayed little bacterial killing, peaking after 4 h with a 1.25-log_10_ CFU/ml decrease in the bacterial burden, but consistently showed no difference in CFU per milliliter compared to the control after 24 h due to extensive regrowth. The 0.5-μg/ml (0.5× MIC) concentration displayed bacterial killing, peaking after 8 h with a 3.6-log_10_ CFU/ml reduction compared to the control; however, inconsistent regrowth was also observed, with only a 1.0-log_10_ CFU/ml difference compared to the control after 24 h. The 1.0-μg/ml (1.0× MIC) concentration displayed more effective bacterial killing activity, particularly at 8 h, with an ∼4.6-log_10_ CFU/ml decrease in the bacterial burden compared to the control, which was sustained to an ∼4.7-log_10_ CFU/ml decrease after 24 h compared to the control. The 2.0-μg/ml (2.0× MIC) concentration of Leu_10_-teixobactin showed very effective bacterial killing activity by causing an ∼7.2-log_10_ CFU/ml decline in the bacterial burden after 24 h compared to the control, with no detectable regrowth. Similarly, the 4.0-μg/ml (4.0× MIC) concentration illustrated very effective killing activity by producing an 8.0-log_10_ CFU/ml reduction compared to the control after 24 h, with no sign of regrowth. The time-kill studies showed that not unlike the data reported previously for native teixobactin (5), the Leu_10_-teixobactin analogue has a time-dependent killing effect; however, at a sub-MIC (i.e., <1.0 μg/ml), extensive regrowth occurs over 24 h.

10.1128/mSystems.00077-20.1 FIG S1 (A) Time-kill curves for various concentrations of Leu_10_-teixobactin against methicillin-resistant S. aureus strain ATCC 700699 (Leu_10_-teixobactin MIC of 1 μg/ml). (B) Time-kill curve for 0.5 μg/ml Leu_10_-teixobactin against methicillin-resistant S. aureus strain ATCC 700699 (Leu_10_-teixobactin MIC of 1 μg/ml) in late exponential phase for the purposes of metabolomics preparation. Data points are plotted as the means ± SD from three independent measurements. Download FIG S1, TIF file, 1.8 MB. Copyright © 2020 Hussein et al. 2020 Hussein et al. This content is distributed under the terms of the Creative Commons Attribution 4.0 International license.

The killing activity of Leu_10_-teixobactin in static time-kill studies was assessed against S. aureus ATCC 700699 in late-exponential-phase growth using a sub-MIC for the purposes of determining appropriate conditions for metabolomics sample preparation, wherein extensive bacterial killing is undesirable (Fig. S1B). The 0.5-μg/ml (0.5× MIC) concentration of Leu_10_-teixobactin showed some bacterial killing, peaking after 3 h with an ∼1.7-log_10_ CFU/ml decrease compared to the control, which stayed consistent after 6 h and was deemed a suitable sub-MIC treatment concentration for the metabolomics experiments.

### Multivariate and univariate analyses of the metabolites affected 1, 3, and 6 h following Leu_10_-teixobactin treatment of S. aureus ATCC 700699.

Under all treatment conditions, a total of 916 putative metabolites were identified, including 54 metabolites in carbohydrate metabolism, 140 metabolites in amino acid metabolism, 37 metabolites in nucleotide metabolism, and 166 metabolites in lipid metabolism. A multivariate data analysis using one-way analysis of variance (ANOVA) followed by principal-component analysis (PCA) was performed to determine the significant metabolites affected 1, 3, and 6 h following Leu_10_-teixobactin treatment (0.5 μg/ml; 0.5× MIC; ≥1-log_2_-fold; *P* ≤ 0.05; false discovery rate [FDR], ≤0.05). The reproducibility for all sample groups was acceptable across all time points (1, 3, and 6 h), where the median relative standard deviations (RSDs) across all time points were 20 to 27% for untreated (control) groups and 18 to 25% for treated samples, consistent with some baseline variability in the dynamics of ordinary bacterial metabolism with and without Leu_10_-teixobactin treatment (Table S1). Notably, the PCA plots showed that the untreated control and Leu_10_-teixobactin-treated samples were significantly separated across all the time points (1, 3, and 6 h) (Fig. S2). Similarly, the heat maps reflected the above-mentioned differences between the Leu_10_-teixobactin-treated groups and the untreated (control) groups (Fig. S3). Notably, the heat maps showed that the metabolite intensities underwent a dramatic decline after Leu_10_-teixobactin treatment compared to the untreated control samples, particularly at 3 h.

10.1128/mSystems.00077-20.2 FIG S2 PCA plots for metabolite levels from S. aureus ATCC 700699 samples treated with Leu_10_-teixobactin at 1 h (i), 3 h (ii), and 6 h (iii). Each data set represents a total of 8 samples of 4 biological replicates under each condition. Red, control (untreated); green, Leu_10_-teixobactin. Download FIG S2, TIF file, 1.8 MB. Copyright © 2020 Hussein et al. 2020 Hussein et al. This content is distributed under the terms of the Creative Commons Attribution 4.0 International license.

10.1128/mSystems.00077-20.3 FIG S3 Heat map profiles of S. aureus ATCC 700699 with hierarchical clustering of all identified metabolites after treatment with Leu_10_-teixobactin at 1, 3, and 6 h. Download FIG S3, TIF file, 2.4 MB. Copyright © 2020 Hussein et al. 2020 Hussein et al. This content is distributed under the terms of the Creative Commons Attribution 4.0 International license.

10.1128/mSystems.00077-20.7 TABLE S1 Data precision of individual metabolomics samples represented as the median relative standard deviation (RSD) for all metabolites of S. aureus ATCC 700699 based on all replicates (*n *= 4) of each group (*n* = 8 for technical replicates of pooled biological quality controls [PBQCs]). Download Table S1, PDF file, 0.1 MB. Copyright © 2020 Hussein et al. 2020 Hussein et al. This content is distributed under the terms of the Creative Commons Attribution 4.0 International license.

Leu_10_-teixobactin treatment perturbed 232 (1 h), 371 (3 h), and 282 (6 h) significant metabolites, most of which were diminished in response to treatment (Fig. S4A). Across all time points, there were 104 overlapping metabolites and 54, 87, and 52 unique metabolites at 1, 3, and 6 h, respectively (Fig. S4B). The classification of the significantly impacted metabolites reveals that amino acids, peptides, lipids, and carbohydrates were largely perturbed (decreased) compared to nucleotide biosynthesis and energy metabolism metabolites, which were less significantly impacted (Fig. S5). This pattern was seen across all time points (Fig. S5A to C).

10.1128/mSystems.00077-20.4 FIG S4 (A) Summary number of significant metabolite changes (≥1.0-log_2_-fold; *P* ≤ 0.05; FDR ≤ 0.05) of S. aureus ATCC 700699 after Leu_10_-teixobactin treatment at 1, 3, and 6 h. (B) Venn diagrams representing the number of metabolites significantly affected by Leu_10_-teixobactin treatment (≥1.0-log_2_-fold; *P* ≤ 0.05; FDR ≤ 0.05) at 1, 3, and 6 h. Download FIG S4, TIF file, 1.8 MB. Copyright © 2020 Hussein et al. 2020 Hussein et al. This content is distributed under the terms of the Creative Commons Attribution 4.0 International license.

10.1128/mSystems.00077-20.5 FIG S5 Total number of significant metabolite changes classified according to metabolite classes after Leu_10_-teixobactin treatment of S. aureus ATCC 700699 at 1 h (A), 3 h (B), and 6 h (C) (≥1.0-log_2_-fold; *P* ≤ 0.05; FDR ≤ 0.05). Note that “Others” denotes cofactor and vitamins, secondary metabolites, and glycan biosynthesis. Download FIG S5, TIF file, 1.8 MB. Copyright © 2020 Hussein et al. 2020 Hussein et al. This content is distributed under the terms of the Creative Commons Attribution 4.0 International license.

### Perturbations of glycerophospholipid and fatty acid metabolism.

**FIG 2:**
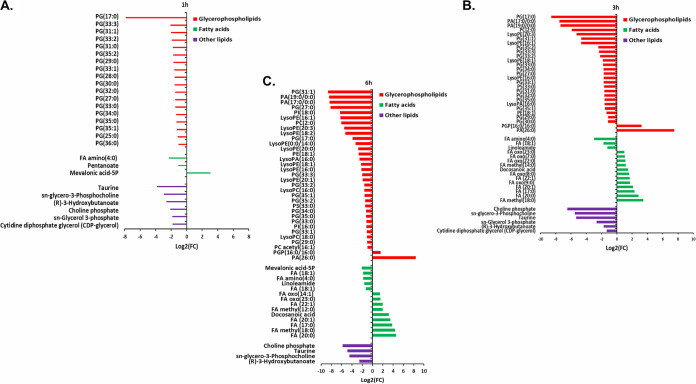
Significantly impacted lipids in S. aureus ATCC 700699 following treatment with Leu_10_-teixobactin at 1 h (A), 3 h (B), and 6 h (C). Putative lipid names are assigned based on accurate mass (≥1.0-log_2_-fold; *P* ≤ 0.05; FDR ≤ 0.05). PE, phosphoethanolamines; PG, glycerophosphoglycerols; PS, glycerophosphoserines; PC, glycerophosphocholines; PA, glycerophosphates; PI, glycerophosphoinositols; PGP, glycerophosphoglycerophosphates; LysoPE, lysophosphatidylethanolamines; LysoPA, lysophosphatidic acid; LysoPC, lysophosphatidylcholines; FA, fatty acids.

At 1 h, Leu_10_-teixobactin treatment caused more perturbations in glycerophospholipid levels than in the levels of fatty acid (FA) and other lipid classes (Fig. 2A). Nevertheless, there was a significant decline in almost all perturbed lipid classes, particularly glycerophospholipids, mainly long-chain glycerophosphoglycerols such as the phosphatidylglycerols PG(31:0), PG(33:0), and PG(35:1). Among the glycerophospholipids, levels of CDP-glycerol, which is an essential precursor involved in bacterial lipid synthesis and teichoic acid biosynthesis (25), decreased significantly (log_2_-fold change [log_2_FC] = −1.8) after Leu_10_-teixobactin treatment at 1 and 3 h (Fig. 2A and B). In the case of the perturbed fatty acids, the treated samples displayed a marked increase in an important intermediate of the mevalonate pathway, namely, mevalonic acid-5 phosphate (5P) (log_2_FC = 3.0) (Fig. 2A). The levels of two other well-known metabolites in bacterial glycerophospholipid biosynthesis, namely, *sn*-glycerol 3-phosphate (log_2_FC = −1.8) and *sn*-glycero-3-phosphocholine (log_2_FC = −2.9), were significantly decreased at 1 h (Fig. 2A).

At 3 h, more changes in glycerophospholipid levels were observed than for fatty acid intermediates (Fig. 2B). There was a significant decline in core intermediates of glycerophospholipid metabolism, including the lysophosphatidylethanolamines LysoPE(16:0) (log_2_FC = −1.7) and LysoPE(16:1) (log_2_FC = −4.6) and the lysophosphatidic acid LysoPA(16:0) (log_2_FC = −1.6). Furthermore, CDP-glycerol levels were also diminished (log_2_FC = −1.3194). On the other hand, there was a significant rise in the fatty acid levels except for FA(18:1) (log_2_FC = −1.7), linoleamide (log_2_FC = −1.2), and FA amino(4:0) (log_2_FC = −2.98). Similar to the perturbation seen at the 1-h time point, albeit to a greater extent, the concentrations of *sn*-glycerol 3-phosphate (log_2_FC = −2.6) and *sn*-glycero-3-phosphocholine (log_2_FC = −5.4) were significantly decreased (Fig. 2B).

Similar patterns were evident at 6 h, wherein levels of glycerophospholipid intermediates displayed dramatic changes (mostly decreases) compared to the fatty acid levels (mainly increases) (Fig. 2C). Among the glycerophospholipids, the levels of LysoPE(16:1) (log_2_FC = −6.2), LysoPE(0:0/14:0) 1-hydroxy-2-myristoyl-*sn*-glycero-3-phosphoethanolamine (log_2_FC = −3.3), and LysoPA(16:0) (log_2_FC = −2.5) decreased, while the levels of the phosphatidylglycerolphosphate PGP(16:0/16:0) 1,2-dipalmitoylphosphatidylglycerol phosphate (log_2_FC = 1.5) and the phosphatidic acid PA(26:0) (log_2_FC = 8.4) increased. Similarly, the levels of the fatty acid metabolites mevalonic acid-5P (log_2_FC = −2.0), FA(18:1) (log_2_FC = −1.8), and linoleamide (log_2_FC = −1.6) significantly decreased at 6 h (Fig. 2C). Consistent with the perturbations seen at the 1- and 3-h time points, the levels of *sn*-glycero-3-phosphocholine (log_2_FC = −4.5) and *sn*-glycerol 3-phosphate (log_2_FC = −1.1) significantly decreased at 6 h (Fig. 2C).

### Perturbations of amino-sugar and sugar-nucleotide metabolism and downstream peptidoglycan (premature peptidoglycan) and teichoic acid biosynthesis.

**FIG 3:**
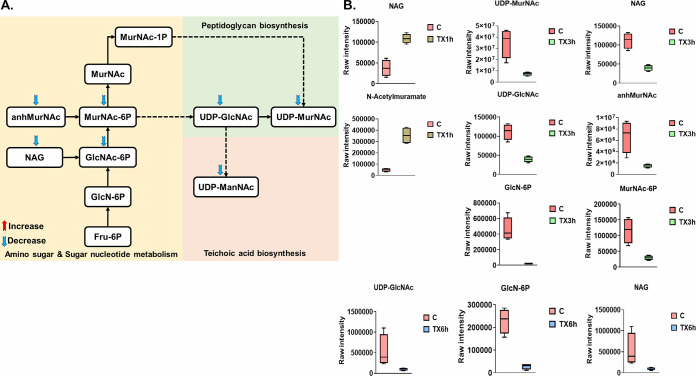
(A) Diagrammatic representation of all significantly impacted amino-sugar and sugar-nucleotide metabolites (3 h) in S. aureus ATCC 700699. (B) Box plots for significantly perturbed metabolites of S. aureus ATCC 700699 following Leu_10_-teixobactin treatment at 1, 3, and 6 h (≥1.0-log_2_-fold; *P* ≤ 0.05; FDR ≤ 0.05). C, control (untreated); TX, Leu_10_-teixobactin.

**FIG 4:**
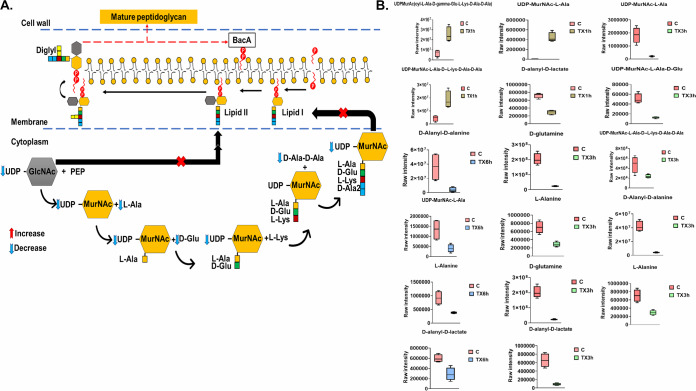
(A) Diagrammatic representation of perturbed peptidoglycan biosynthesis after Leu_10_-teixobactin treatment (3 h). (B) Box plots for significantly perturbed intermediates of peptidoglycan biogenesis at 1, 3, and 6 h (≥1.0-log_2_-fold; *P* ≤ 0.05; FDR ≤ 0.05). C, control (untreated); TX, Leu_10_-teixobactin; PEP, phosphoenolpyruvate.

**FIG 5:**
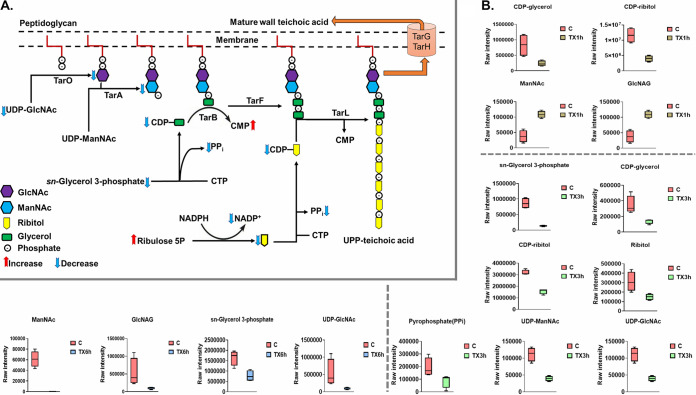
(A) Perturbed teichoic acid metabolism following Leu_10_-teixobactin treatment (3 h). (B) Box plots of the significantly perturbed precursors of teichoic acid formation after Leu_10_-teixobactin treatment at 1, 3, and 6 h (≥1.0-log_2_-fold; *P* ≤ 0.05; FDR ≤ 0.05). C, control (untreated); TX, Leu_10_-teixobactin; UPP, undecaprenyl-pyrophosphate.

Significant impacts on amino-sugar and sugar-nucleotide metabolism, both of which are essential pools that feed key precursors into the fundamental metabolic pathways of peptidoglycan and cell wall teichoic acid biogenesis, were observed 1, 3, and 6 h after treatment (Fig. 3A and B). In line with the primary mode of action of teixobactin, Leu_10_-teixobactin caused a significant perturbation of intermediates involved in all initial steps of lipid I and lipid II biosynthesis of peptidoglycan and the main precursors of wall teichoic acid (WTA) formation (teichoic acid lipid III) across all time points (1, 3, and 6 h) (Fig. 4A and B and Fig. 5A and B). Leu_10_-teixobactin treatment resulted in minimal changes to the above-mentioned pathways at 1 h, while a greater influence was remarkably noted at later time points, in particular at 3 h (Fig. 3A, Fig. 4A, and Fig. 5A).

Minor effects were observed at 1 h, wherein the levels of only two amino-sugar and sugar-nucleotide intermediates (the main peptidoglycan building blocks) showed substantial increases, including *N*-acetylmuramate (MurNAc) and *N*-acetyl-d-glucosamine (NAG or GlcNAc) (>1.5-log_2_-fold; *P* ≤ 0.05; FDR ≤ 0.05) (Fig. 3B). Likewise, Leu_10_-teixobactin treatment produced a significant impact on the levels of three key components involved in peptidoglycan biosynthesis, including UDP-*N*-acetylmuramoyl-l-alanine (UDP-MurNAc-l-Ala), UDP-MurAc(oyl-l-Ala-d-γ-Glu-l-Lys-d-Ala-d-Ala), and UDP-*N*-acetylmuramoyl-l-alanyl-d-glutamyl-l-lysyl-d-alanyl-d-alanine (UDP-MurNAc-l-Ala-γ-d-Glu-l-Lys-d-Ala-d-Ala) (>1.5-log_2_-fold; *P* ≤ 0.05; FDR ≤ 0.05) (Fig. 4B). Consequently, the Leu_10_-teixobactin-treated samples manifested a significant overrepresentation in the biogenesis of WTA at 1 h wherein the levels of eight crucial components underwent a marked perturbation, including *N-*acetyl-d-mannosamine (ManNAc) (log_2_FC = 1.5), GlcNAc (log_2_FC = 1.5), CDP-ribitol (log_2_FC = −1.5), CDP-glycerol (log_2_FC = −1.8), *sn*-glycerol 3-phosphate (log_2_FC = 1.5), ribulose 5-phosphate (log_2_FC = −1.1), pyrophosphate (PP_i_) (log_2_FC = −4.9), and CMP (log_2_FC = −1.5) (Fig. 5B).

Notably, at 3 h, Leu_10_-teixobactin treatment led to marked decreases in the levels of six crucial amino-sugar and sugar-nucleotide intermediates, namely, NAG, MurNAc, d-glucosamine 6-phosphate (GlcN-6P), 1,6-anhydro-*N*-acetylmuramate (anhMurNAc), UDP-GlcNAc, and *N*-acetylmuramate 6-phosphate (MurNAc-6P) (greater than −1.5-log_2_-fold; *P* ≤ 0.05; FDR ≤ 0.05) (Fig. 3A and B). Accordingly, dramatic declines in the levels of peptidoglycan biosynthetic precursor metabolites were observed, namely, UDP-MurNAc-l-Ala, UDP-MurNAc-l-Ala-γ-d-Glu-l-Lys-d-Ala-d-Ala, UDP-*N*-acetylmuramoyl-l-alanyl-d-glutamate (UDP-MurNAc-l-Ala-d-Glu), d-alanyl-d-alanine, l-alanine, and d-glutamine (greater than −1.5-log_2_-fold; *P* ≤ 0.05; FDR ≤ 0.05) (Fig. 4A and B). A more profound inhibitory effect on WTA biosynthesis was also observed following Leu_10_-teixobactin treatment at 3 h, at which point 11 fundamental building blocks of WTA underwent substantial declines in their abundances, including ManNAc, UDP-ManNAc, GlcNAc, CDP-ribitol, CDP-glycerol, and ribitol (greater than −1.0-log_2_-fold; *P* ≤ 0.05; FDR ≤ 0.05) (Fig. 5A and B). There were also reductions in the levels of ribulose 5-phosphate (log_2_FC = −4.9) and CMP (log_2_FC = −4.8) 3 h after Leu_10_-teixobactin treatment.

At 6 h, there were marginal differences in the above-mentioned perturbations seen at 3 h. Notably, three essential precursors of amino-sugar and nucleotide-sugar metabolism were depleted, namely, GlcN-6P, UDP-GlcNAc, and NAG (greater than −2.0-log_2_-fold; *P* ≤ 0.05; FDR ≤ 0.05) (Fig. 3B). Downstream peptidoglycan biosynthesis was severely perturbed, wherein the concentrations of three fundamental metabolites declined considerably following treatment; these were d-alanyl-d-alanine, UDP-MurNAc-l-Ala, and l-alanine (greater than or equal to −1.0-log_2_-fold; *P* ≤ 0.05; FDR ≤ 0.05) (Fig. 4B). Similarly, WTA was considerably perturbed by Leu_10_-teixobactin treatment, wherein the levels of six intermediates decreased dramatically, including ManNAc, GlcNAc, UDP-ManNAc, *sn*-glycerol 3-phosphate, pyrophosphate, and NADP^+^ (Fig. 5B).

Interestingly, the abundance of d-alanyl-d-lactate, an essential regulatory intermediate of vancomycin resistance, was significantly reduced following Leu_10_-teixobactin treatment across all time points (1, 3, and 6 h) (log_2_FC = −1.3, −2.9, and −1.1, respectively) (Fig. 4B).

### Perturbations of histidine metabolism.

**FIG 6:**
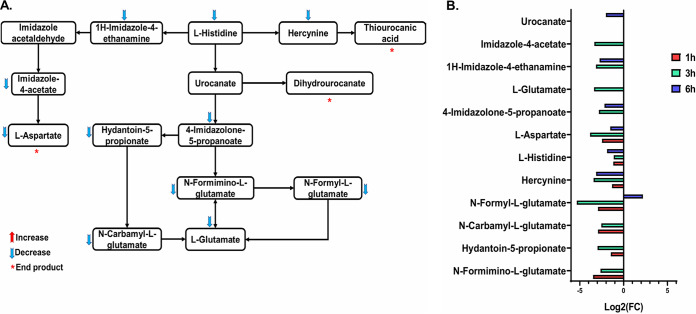
(A) Schematic representation of impacted histidine metabolism in S. aureus ATCC 700699 after Leu_10_-teixobactin treatment (3 h). (B) Fold change chart for the significantly impacted metabolites from histidine metabolism in S. aureus ATCC 700699 treated with Leu_10_-teixobactin at 1, 3, and 6 h (≥1.0-log_2_-fold; *P* ≤ 0.05; FDR ≤ 0.05).

The histidine biosynthetic pathway contains several fundamental intermediates and enzymatic steps, which constitute a critical link between amino acid, purine, and thiamine biosynthesis (26). Leu_10_-teixobactin treatment caused significant perturbations in several metabolite intermediates involved in histidine biosynthesis and degradation across all time points albeit most noticeably at 3 h (Fig. 6A and B). This effect on the histidine degradation pathway potentially reflects the upregulation of the l-glutamate synthesis pathway, which is known to be part of the bacterial response to stress (27). A large number of metabolites involved in histidine metabolism were significantly impacted by Leu_10_-teixobactin at 1 h, namely, l-histidine (log_2_FC = −1.2), *N*-formyl-l-glutamate (log_2_FC = −2.9), *N*-carbamyl-l-glutamate (log_2_FC = −2.9), hydantoin-5-propionate (log_2_FC = −1.4), *N*-formimino-l-glutamate (log_2_FC = −3.5), l-aspartate (log_2_FC = −2.4), and hercynine (log_2_FC = −1.3) (Fig. 6B). At 3 h, the levels of 11 crucial histidine metabolites were markedly altered by Leu_10_-teixobactin treatment (Fig. 6A and B). These intermediates were l-histidine (log_2_FC = −1.1), *N*-formyl-l-glutamate (log_2_FC = −5.3), *N*-carbamyl-l-glutamate (log_2_FC = −2.5), hydantoin-5-propionate (log_2_FC = −2.9), *N*-formimino-l-glutamate (log_2_FC = −2.6), l-aspartate (log_2_FC = −3.8), hercynine (log_2_FC = −3.4), 4-imidazolone-5-propanoate (log_2_FC = −2.8), 1H-imidazole-4-ethanamine (log_2_FC = −3.1), imidazole-4-acetate (log_2_FC = −3.3), and l-glutamate (log_2_FC = −3.4) (Fig. 6A and B). Similar patterns, although to a lesser extent, were evident at 6 h, wherein six histidine biosynthetic metabolites were significantly reduced in the Leu_10_-teixobactin-treated samples, including l-histidine (log_2_FC = −1.9), l-aspartate (log_2_FC = −1.5), hercynine (log_2_FC = −3.1), 4-imidazolone-5-propanoate (log_2_FC = −2.2), 1H-imidazole-4-ethanamine (log_2_FC = −2.7), and urocanate (log_2_FC = −2.0). In contrast to the 1- and 3-h time points, the level of one particular intermediate, *N*-formyl-l-glutamate, which represents the last intermediate before glutamate (the final product of the histidine degradation pathway), was significantly elevated (log_2_FC = 2.2) after Leu_10_-teixobactin treatment at 6 h (Fig. 6B).

### Perturbations of arginine metabolism and the closely interrelated tricarboxylic acid cycle.

**FIG 7:**
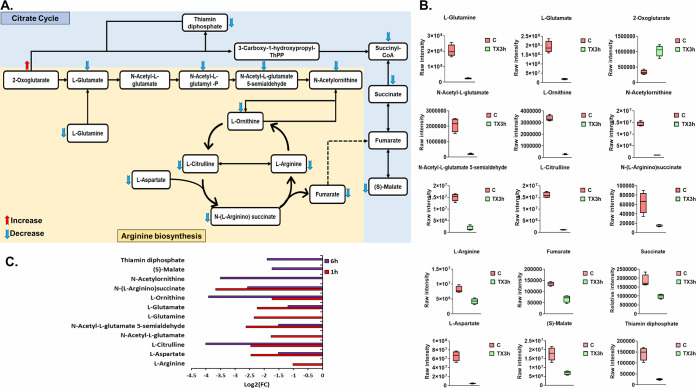
(A) Schematic pathway diagram depicting arginine metabolism and the interrelated TCA cycle in Leu_10_-teixobactin-treated S. aureus ATCC 700699 at 3 h. (B) Box-and-whisker plots for significantly affected components of arginine metabolism and the downstream TCA cycle following Leu_10_-teixobactin treatment at 3 h. (C) Fold change chart for the significantly impacted metabolites from arginine metabolism and the interrelated TCA cycle in S. aureus ATCC 700699 treated with Leu_10_-teixobactin at 1 and 6 h (≥1.0-log_2_-fold; *P* ≤ 0.05; FDR ≤ 0.05). C, control (untreated); TX, Leu_10_-teixobactin; ThPP, thiamine pyrophosphate.

The impacts of Leu_10_-teixobactin on arginine metabolism and the interrelated tricarboxylic acid (TCA) cycle were significant across all time points, particularly at 3 h (Fig. 7A and B). The TCA cycle is essential for bacterial cellular respiration and supplies various intermediates, such as succinate and citrate, which are required for other key metabolic processes (28). Notably, the disruption of amino acid pathways, such as arginine metabolism, has developed as a new method to control bacterial infections and subvert pathogenesis (29). At 1 h, the levels of nine arginine metabolism intermediates were decreased, including l-arginine, l-aspartate, l-glutamate, l-glutamine, l-citrulline, l-ornithine, *N*-acetyl-l-glutamate, *N*-(l-arginino)succinate, and *N*-acetyl-l-glutamate 5-semialdehyde (greater than or equal to −1.0-log_2_-fold; *P* ≤ 0.05; FDR ≤ 0.05) (Fig. 7C). A marginal impact on the TCA cycle was evident at 1 h, with a significant drop seen only in the levels of one TCA cycle intermediate, namely, (*S*)-malate (log_2_FC = −1.8) (Fig. 7C). At 3 h, the levels of 11 fundamental metabolites involved in arginine metabolism showed marked reductions following Leu_10_-teixobactin treatment (except for 2-oxoglutarate, which was elevated [log_2_FC = 1.6]): l-glutamine (log_2_FC = −3.2), l-glutamate (log_2_FC = −3.4), l-ornithine (log_2_FC = −3.6), and l-arginine (log_2_FC = −0.9) (Fig. 7A and B). The interrelated TCA cycle was also substantially perturbed at 3 h, wherein the concentrations of four crucial TCA cycle intermediates decreased after Leu_10_-teixobactin treatment, including fumarate, succinate, (*S*)-malate, and thiamine diphosphate (greater than or equal to −0.59-log_2_-fold; *P* ≤ 0.05; FDR ≤ 0.05) (Fig. 7A and B).

Similarly, the inhibitory effect of Leu_10_-teixobactin on both pathways was perpetuated at 6 h postexposure, with a maximum peak effect at around 3 h. At 6 h, seven arginine metabolism intermediates were considerably depleted (greater than or equal to −1.0-log_2_-fold; *P* ≤ 0.05; FDR ≤ 0.05), including l-glutamate, l-aspartate, l-citrulline, l-ornithine, *N*-acetylornithine, *N*-(l-arginino)succinate, and *N*-acetyl-l-glutamate 5-semialdehyde (Fig. 7C). This inhibitory effect was accompanied by substantial decreases in the levels of two essential precursors in the TCA cycle, namely, (*S*)-malate and thiamine diphosphate (Fig. 7C).

### Perturbations of pantothenate and coenzyme A biosynthesis.

**FIG 8:**
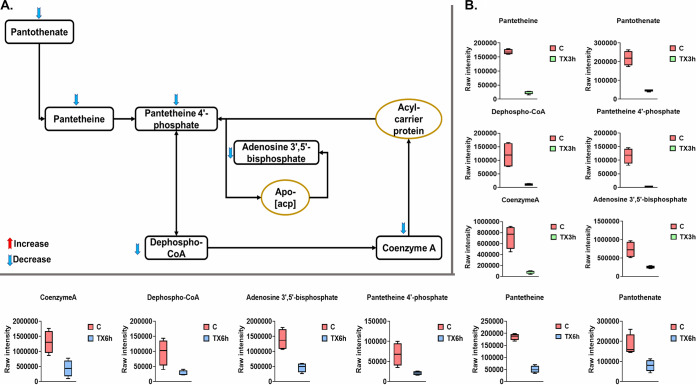
(A) Significantly impacted pantothenate and CoA biosynthesis in S. aureus ATCC 700699 treated with Leu_10_-teixobactin at 3 h. (B) Box-and-whisker plots for the significantly perturbed metabolites from pantothenate and CoA biosynthesis in S. aureus ATCC 700699 treated with Leu_10_-teixobactin at 3 and 6 h (≥1.0-log_2_-fold; *P* ≤ 0.05; FDR ≤ 0.05). C, control (untreated); TX, Leu_10_-teixobactin; acp, acyl carrier protein.

Leu_10_-teixobactin treatment significantly altered the levels of metabolites related to pantothenate and coenzyme A (CoA) biosynthesis largely at 3 and 6 h (Fig. 8A and B). This effect may be related to the above-mentioned impact of Leu_10_-teixobactin on membrane biogenesis and the TCA cycle, as CoA and its derivative phosphopantetheine are vital for enzymes involved in the synthesis of phospholipids, fatty acid synthesis and degradation, and the oxidation of pyruvate in the TCA cycle (30). At 3 h, the levels of six intermediates of pantothenate and coenzyme A biosynthesis were significantly decreased after Leu_10_-teixobactin exposure, namely, pantothenate, pantetheine, pantetheine 4′-phosphate, dephospho-CoA, adenosine 3′,5′-bisphosphate, and CoA (greater than or equal to −1.5-log_2_-fold; *P* ≤ 0.05; FDR ≤ 0.05) (Fig. 8A and B). Similarly, Leu_10_-teixobactin treatment caused dramatic decreases in the levels of the pantothenate and coenzyme A intermediates at 6 h (Fig. 8B), wherein pantothenate, pantetheine, pantetheine 4′-phosphate, dephospho-CoA, adenosine 3′,5′-bisphosphate, and CoA (greater than or equal to −1.5-log_2_-fold; *P* ≤ 0.05; FDR ≤ 0.05) were greatly diminished relative to the untreated control. These perturbations were not observed at the 1-h time point.

### Conclusions.

**FIG 9:**
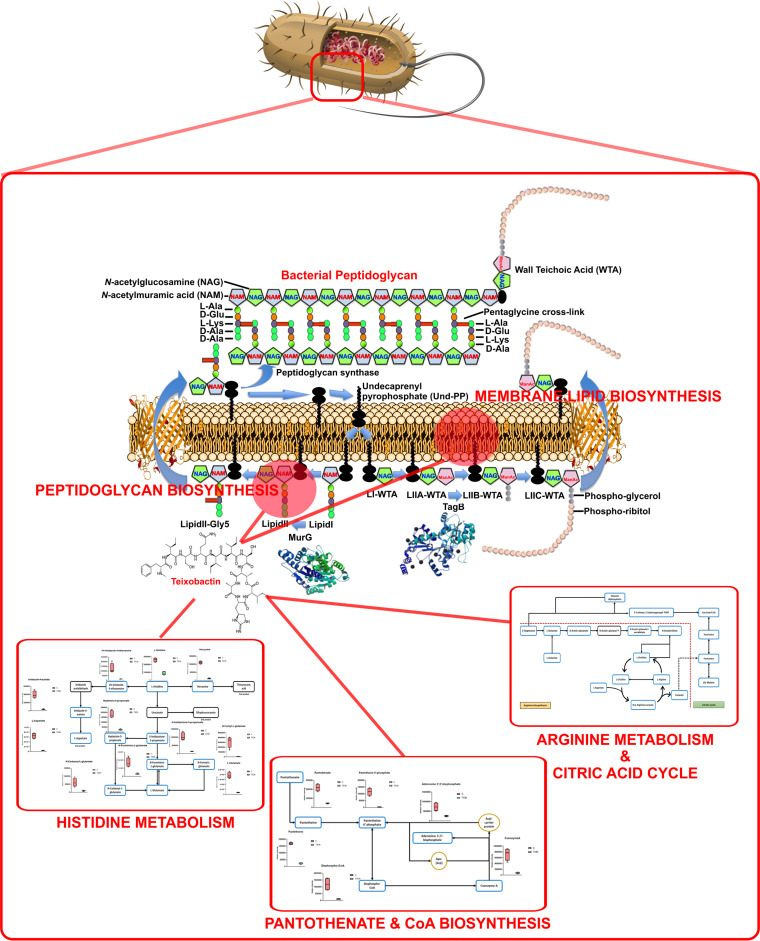
Schematic diagram summarizing the key metabolic changes in S. aureus ATCC 700699 in response to Leu_10_-teixobactin treatment.

MRSA represents a critical challenge to human health worldwide. Due to the rapid emergence of resistance to currently available antibiotics, there is an urgent need to develop new antimicrobials with novel mechanisms of action. Teixobactin is the first antimicrobial compound with a novel mode of action discovered in over a decade. More importantly, to date, it remains the first “resistance-resistant” compound ever discovered against MRSA; however, its mechanisms of action are not well understood. In the present study, we employed comparative global metabolomics to elucidate the key pathways associated with Leu_10_-teixobactin killing of a MRSA strain, S. aureus ATCC 700699 (Fig. 9). The metabolomes of S. aureus ATCC 700699 cells were systematically compared 1, 3, and 6 h following treatment with Leu_10_-teixobactin (0.5 μg/liter, i.e., 0.5× MIC). Leu_10_-teixobactin significantly perturbed bacterial membrane lipids and cell wall biosynthesis (peptidoglycan and teichoic acid) at 1 h, reflecting its initial activity on the cell envelope. In a time-dependent fashion and to a greater extent (concordant with its time-dependent antibacterial killing action), Leu_10_-teixobactin also perturbed the levels of key intermediates in the pathways of arginine metabolism and the interrelated TCA cycle, histidine metabolism, amino-sugar and nucleotide-sugar metabolism, and pantothenate and CoA biosynthesis. More importantly, Leu_10_-teixobactin significantly reduced the main intermediate (d-alanyl-d-lactate) involved in the mechanism of vancomycin resistance in S. aureus. This study provides novel mechanistic insight to help support the development of teixobactin as an antibacterial drug for the treatment of MDR Gram-positive infections.

## MATERIALS AND METHODS

### Stock solutions and bacterial isolates.

Leu_10_-teixobactin was chemically synthesized as described below, and solutions were prepared by dissolving the compound in dimethyl sulfoxide (DMSO) and filtered through 0.22-μm syringe filters (Sartorius, Australia). Five strains of Staphylococcus aureus were used in these experiments. These included 2 strains of methicillin-resistant S. aureus (MRSA), S. aureus ATCC 700698 and S. aureus ATCC 700699; 2 strains of vancomycin-intermediate S. aureus, S. aureus JKD6008 and S. aureus JKD6009; and the susceptible strain S. aureus ATCC 29213. The isolates were stored in tryptone soy broth (Oxoid) with 20% glycerol (Ajax Finechem, Seven Hills, NSW, Australia) in cryovials at −80°C. Before use, the strains were subcultured onto nutrient agar plates (Media Preparation Unit, The University of Melbourne, Melbourne, VIC, Australia) for 24 h.

### MIC measurements.

MICs of Leu_10_-teixobactin were determined for all isolates using broth microdilution in Mueller-Hinton broth (MHB; Oxoid, England) supplemented with 0.1% Tween 80 to avoid the adhesion of the compound to the 96-well microtiter plates (19). Stock solutions of Leu_10_-teixobactin were prepared in dimethyl sulfoxide (Sigma-Aldrich) immediately before each experiment. The solution was then serially diluted in MHB medium to create a range of solutions from 0.125 to 128 μg/ml, which were then added to 96-well microtiter plates (Techno Plas). All tested strains were exposed to a final concentration of DMSO (2.5%, vol/vol) that had no inhibitory effect on their growth. The subculture-isolated colonies were added to 0.9% saline using a sterile swab and then measured using a McFarland meter (bioMérieux) to reach a 0.5 McFarland turbidity standard. The culture was then diluted 100-fold to give an approximate starting inoculum of 10^6^ CFU/ml in each well of the 96-well microtiter plates. The plates were then incubated at 37°C for 18 to 24 h. MICs were determined as the lowest concentrations that inhibited the visible growth of the bacteria.

### Static time-kill studies.

Time-kill studies with Leu_10_-teixobactin were conducted against S. aureus ATCC 70699. Leu_10_-teixobactin concentrations of 0.25, 0.5, 1.0, 2.0, and 4.0 μg/ml dissolved in DMSO were tested at exposure times of 0, 0.5, 1, 2, 4, 8, and 24 h. A culture grown overnight was prepared by inoculating one colony of the isolate from a fresh subculture in 10 ml MHB (Oxoid) in 50-ml Falcon tubes (Thermo Fisher, Australia) and incubated in a shaking water bath at 37°C for 16 h. After incubation overnight, 100 μl of the culture was added to 10 ml MHB and then incubated for 2 h to obtain a log-phase culture. Next, 200 μl of the log-phase culture was added to 20 ml of fresh MHB in glass tubes (Kimax) to obtain an initial inoculum of approximately 10^6^ CFU/ml. Before each experiment, stock solutions of Leu_10_-teixobactin were prepared in DMSO. Leu_10_-teixobactin was added to these glass tubes to give the desired final concentrations, leaving one tube as a drug-free control. At each time point, 200-μl samples were removed from each tube and serially diluted in 0.9% saline in 5-ml yellow tubes (Techno Plas), and 50 μl of the resultant bacterial cell suspension was spirally plated onto nutrient agar plates. The nutrient agar plates were incubated at 37°C for 24 h, and the colonies on each plate were then counted to determine the CFU per milliliter of each sample. Time-kill curves were then constructed as the time versus the natural logarithm of the CFU per milliliter.

### Metabolomics sample preparation.

An untargeted metabolomics study was carried out to explore the mechanism(s) of action of Leu_10_-teixobactin against S. aureus ATCC 700699 using a treatment concentration of 0.5 μg/ml (0.5× MIC). Samples were taken and analyzed at the 1-, 3-, and 6-h time points in 4 biological replicates. A culture grown overnight was prepared by inoculating three colonies into 100 ml MHB in 250-ml conical flasks (Pyrex) and incubating the colonies in an incubator shaker (Bioline) at 37°C at 180 rpm for 16 h. After incubation overnight, log-phase cells were prepared in fresh MHB and then incubated for 2 h at 37°C at 180 rpm to log phase with a starting bacterial inoculum of 10^8^ CFU/ml. A stock solution of Leu_10_-teixobactin prepared beforehand was then added to a flask to give a desired concentration of 0.5 μg/ml, leaving another flask as an antibiotic-free control for each replicate. The flasks were then further incubated at 37°C with shaking at 180 rpm. At each time point, 15-ml samples were transferred to 50-ml Falcon tubes (Thermo Fisher, Australia) for quenching, and the optical density reading at 600 nm (OD_600_) was then measured and normalized to the pretreatment level of approximately 0.5 with fresh MHB. Samples were then centrifuged at 3,220 × *g* at 4°C for 10 min, and the supernatants were removed subsequently. The pellets were stored at −80°C, awaiting metabolite extraction.

### Metabolomics metabolite extraction.

The bacterial pellets were washed in 1 ml of 0.9% saline and then centrifuged again at 3,220 × *g* at 4°C for 5 min to remove residual extracellular metabolites and medium components. The pellets were then washed and resuspended to be mixed with a cold extraction solvent (chloroform-methanol-water at 1:3:1, vol/vol) containing 1 μM each the internal standards 3-[(3-cholamidopropyl)-dimethylammonio]-1-propanesulfonate (CHAPS), *N*-cyclohexyl-3-aminopropanesulfonic acid (CAPS), piperazine-*N*,*N*′-bis(2-ethanesulfonic acid) (PIPES), and Tris. The samples were then frozen in liquid nitrogen, thawed on ice, and vortexed to release the intracellular metabolites. Next, the samples were transferred to 1.5-ml Eppendorf tubes and centrifuged at 14,000 × *g* at 4°C for 10 min to get rid of any particulate matter. Finally, 200 μl of the supernatant was taken out and transferred into an injection vial for liquid chromatography-mass spectrometry (LC-MS) analysis. An equal volume of each sample was combined and used as a quality control (QC) sample.

### Data processing, bioinformatics, and statistical analyses.

An Excel interface, namely, IDEOM, was used to convert the LC-MS raw data into annotated and hyperlinked metabolites (31). Initially, the LC-MS raw data were converted to mzXML files using freely available ProteoWizard software. Next, the extracted mzXML files were immediately processed through XCMS to pick peaks and generate peakML files (32). The Mzmatch.R tool was ultimately utilized for filtering and alignment of peaks using a minimum detectable intensity of 100,000, an RSD of <0.5 (reproducibility), and a peak shape (codadw) of >0.8. Mzmatch was also used to retrieve missing peaks and annotate the related peaks. Default IDEOM parameters were employed to remove common sources of noise, including chromatographic peak shoulders, irreproducible peaks, background, or contaminant signals. The loss or gain of a proton was corrected in negative or positive electrospray ionization (ESI) mode, respectively, followed by a data-dependent polynomial mass recalibration step (2 ppm) for the putative metabolites. Putative metabolite identification was obtained by matching the accurate mass and retention time of observed peaks to metabolites in the inbuilt database, which incorporated the Kyoto Encyclopedia of Genes and Genomes (KEGG), MetaCyc, and LIPIDMAPS databases, using preference for bacterial metabolites profiled in EcoCyc. The retention times of authentic standards were used to verify the identified metabolite. The raw peak intensity was used to quantify each metabolite. Statistical analysis was performed using the free online tool MetaboAnalyst 3.0. In brief, the exported table of putative metabolites with a median RSD of ≤0.2 (20%) within the QC group and a confidence level of ≥5 was uploaded to MetaboAnalyst. Data with >50% missing values were removed, and the remaining missing values were replaced by a small value (half the minimum positive value in the original data) (33). Data were filtered using the interquartile range (IQR), normalized by the median, log_2_ transformed, and autoscaled (mean centered and divided by the standard deviation [SD] of each variable). One-way ANOVA and Fisher’s least square difference (LSD) test were used to identify the significantly perturbed metabolites between the treatment and control groups. Statistically significant metabolites were selected using a false discovery rate of ≤0.05 for one-way ANOVA and a *P* value of ≤0.05 for Fisher’s LSD test. The pathway modules were constructed by uploading the KEGG compound numbers of significantly impacted metabolites into KEGG mapper.

### Synthesis of Leu_10_-teixobactin. (i) Synthesis of the C-terminal macrocyclic tetrapeptide (compound 3).

2-Chlorotrityl resin (2.47 g; 2 mmol) was solvated with dichloromethane (DCM) in a 50-ml tube, followed by the addition of a solution of Fmoc (9-fluorenylmethoxy carbonyl)-Ala-OH (686 mg; 2.2 mmol) in DCM (30 ml) (see Fig. S6 in the supplemental material). *N*,*N*-diisopropylethylamine (DIEA) (2.088 ml; 12 mmol) was then added, and the slurry was agitated. After 2 h, methanol (5 ml) was poured in, and the mixture was agitated for a further 30 min. The resin was then filtered in a 25-ml fritted syringe, treated with 20% piperidine in dimethyl formamide (DMF) followed by the addition of Boc-d-Thr-OH (482 mg; 2.2 mmol), and activated with Oxyma pure (312 mg; 2.2 mmol) and *N*,*N*′-diisopropylcarbodiimide (DIC) (346 μl; 2.2 mmol) in DMF. After 1 h, the resin was filtered, and Fmoc-Ile-OH (1.416 g; 4 mmol) activated with DIC (628 μl; 4 mmol) in DCM was added. 4-Dimethylaminopyridine (DMAP) (48 mg; 0.4 mmol) was then added to catalyze ester bond formation. After 2 h, the resin was filtered, and the Fmoc group was cleaved with 20% piperidine in DMF, followed by the addition of Fmoc-l-Leu-OH (778 mg; 2.2 mmol) with (1-cyano-2-ethoxy-2-oxoethylidenaminooxy)dimethylamino-morpholino-carbenium hexafluorophosphate (COMU) (942 mg; 2.2 mmol) and DIEA (766 μl; 4.4 mmol) in DMF. After coupling, the resin was again treated with the piperidine solution to cleave the final Fmoc group. After cleavage with 1% trifluoroacetic acid (TFA) in DCM, the solvent was removed *in vacuo*, followed by resolubilization in acetonitrile-water and lyophilization. A total of 855 mg of crude product was obtained. Two hundred milligrams was isolated via high-performance liquid chromatography (HPLC) to generate 102 mg of purified product. For the cyclization reaction, *N*-(3-dimethylaminopropyl)-*N*′-ethylcarbodiimide hydrochloride (EDC-HCl) (93 mg; 0.484 mmol) and DIEA (141 μl; 0.810 mmol) were added to neat DCM (81 ml). The linear peptide precursor (102 mg; 0.162 mmol) was slowly added over 15 min, and the reaction mixture was stirred for 4 h and monitored via LC-MS. Note that cyclization can also be performed with the crude linear precursor. The solvent was washed with brine and then removed *in vacuo*. TFA (5 ml) was then added, and the reaction mixture was stirred for 30 min. After aspiration with N_2_, the product was solubilized and purified via reverse-phase HPLC (RP-HPLC) purification using HCl buffers (20 to 80% buffer B over 60 min; C_18_). The clean fractions were pooled and lyophilized. A total of 49.0 mg was recovered (70%).

10.1128/mSystems.00077-20.6 FIG S6 (A) C-terminal macrocycle. (i) Fmoc-l-Ile-OH, DIC, DMAP, and DCM for 2 h; (ii) Fmoc SPPS; (iii) 1% TFA and DCM; (iv) 3 eq EDC-HCl, 5 eq DIEA, and DCM at 4 mM for 4 h; (v) 1% H_2_O and TFA for 10 min; (vi) RP-HPLC. (B) Leu_10_-teixobactin. (vii) 3 eq COMU, 5 eq DIEA, and 1,4-dioxane at 60°C for 2 h; (viii) 1% TIPS, 2% H_2_O, and TFA for 2 h; (ix) RP-HPLC. Download FIG S6, TIF file, 2.6 MB. Copyright © 2020 Hussein et al. 2020 Hussein et al. This content is distributed under the terms of the Creative Commons Attribution 4.0 International license.

### (ii) Synthesis of protected teixobactin (1-7) peptide residues (compound 4).

2-Chlorotrityl resin (1.235 g; 1 mmol) was solvated with DCM in a 50-ml tube, followed by the addition of a solution of Fmoc-l-Ile-l-Ser(ψ(Me,Me)pro)-OH (529 mg; 1.1 mmol) in DCM (20 ml). DIEA (1.044 ml; 6 mmol) was then added, and the slurry was agitated for 2 h. Methanol (5 ml) was then added, and the mixture was agitated for a further 30 min. The resin was then filtered in a 25-ml fritted syringe, followed by Fmoc solid-phase peptide synthesis (SPPS) using 1:1 equivalents of the reagent. After peptide assembly, the resin-bound peptide was cleaved with 1% TFA in DCM (100 ml), followed by two brine washes. The solvent was then removed *in vacuo*, and the oily residue was resolubilized with an acetonitrile-water mixture and lyophilized. A total of 960 mg of crude peptide was recovered (76% yield).

### (iii) Synthesis of Leu_10_-teixobactin (compound 6).

Compound 4 (108 mg; 0.086 mmol) was dissolved in 1,4-dioxane (∼4 ml) in a 25-ml round-bottom flask (RBF). COMU (74 mg; 0.173 mmol) was added, followed by DIEA (40 μl; 0.230 mmol). Compound 3 (25 mg; 0.057 mmol) was dissolved in 1,4-dioxane (1 ml) separately, and DIEA (10 μl; 0.057 mmol) was added. This solution was then added to the RBF, and the reaction mixture was heated to 60°C and stirred for 3 h to generate compound 5. The reaction was monitored via LC-MS. The solvent was then removed *in vacuo*, and the oily residue was redissolved in ethyl acetate (EtOAc) (10 ml), followed by two brine washes (2 washes with 10 ml each). The solvent was again removed *in vacuo*, followed by the addition of trifluoroacetic acid-triisopropyl silane-H_2_O (TFA-TIPS-H_2_O) (96:2:2). The reaction mixture was transferred to a 50-ml tube and agitated for 2 h, followed by aspiration with N_2_ to reduce the volume to ∼1 ml. Diethyl ether (40 ml) was then added, and the mixture was cooled with ice. After centrifugation, the supernatant was removed, and the precipitate was air dried, followed by solubilization in a mixture of acetonitrile-water, LC-MS analysis, and HPLC purification. The clean fractions were pooled and lyophilized. A total of 13.1 mg was recovered (17% yield). Analysis via high-resolution mass spectrometry was as follows, where exp is experimental and th is theoretical: monoisotopic [M + H^+^]^+^_exp_ = 1,201.724 ([M + H^+^]^+^_th_ = 1,201.719).

### Data availability.

The metabolomic data set is available at MetaboLights under the study identifier MTBLS1569.
