# Supplementary figures and images for: The Killing Mechanism of Teixobactin against Methicillin-Resistant Staphylococcus aureus: an Untargeted Metabolomics Study
Source: mSystems. 2020 May 26;5(3):e00077-20. doi: 10.1128/mSystems.00077-20 (PMC7253363; doi:10.1128/mSystems.00077-20)

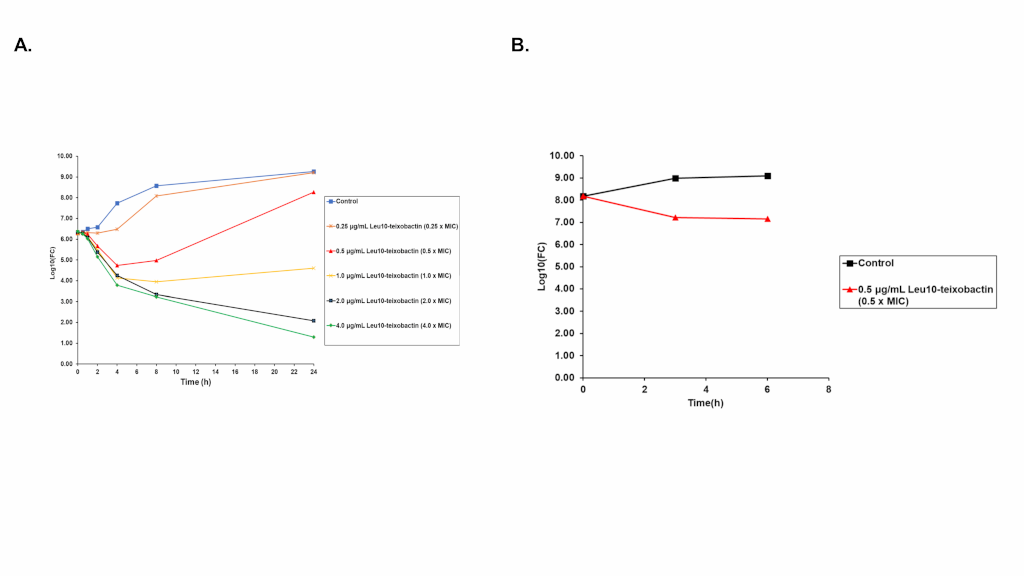

Supplement: FIG S1 [file mSystems.00077-20-sf001.tif]

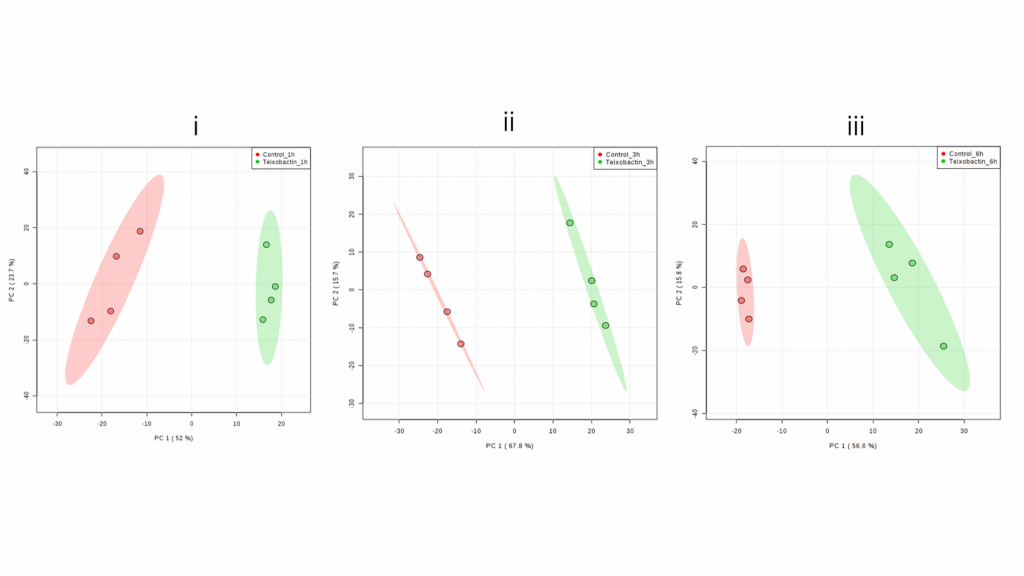

Supplement: FIG S2 [file mSystems.00077-20-sf002.tif]

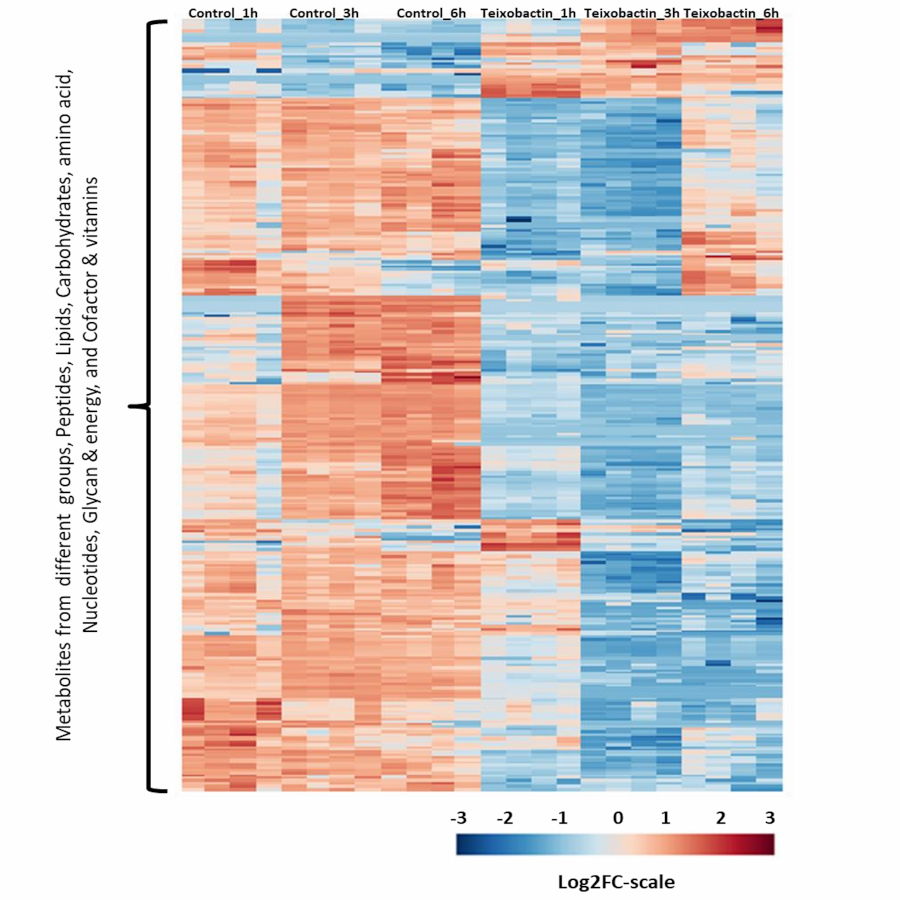

Supplement: FIG S3 [file mSystems.00077-20-sf003.tif]

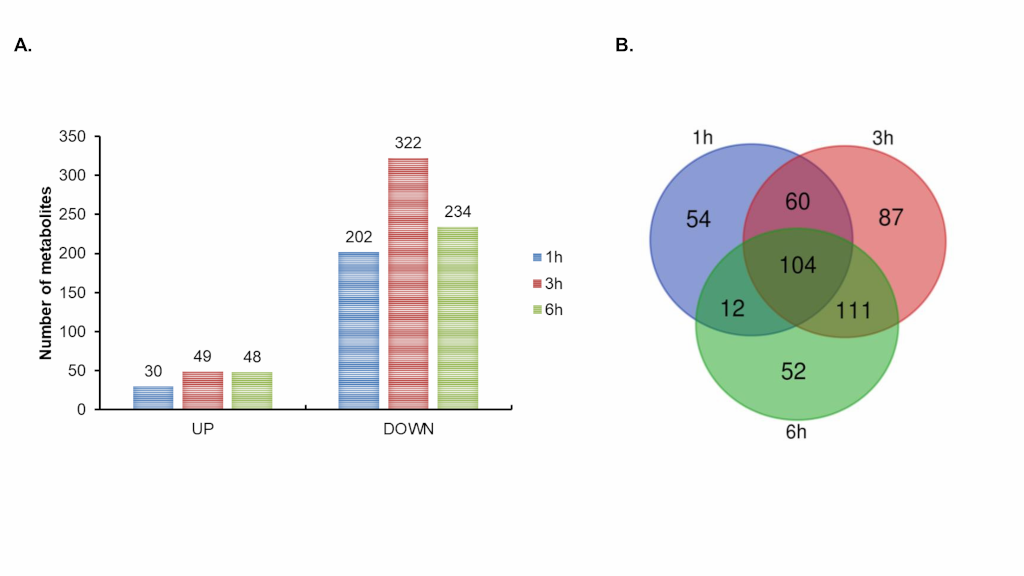

Supplement: FIG S4 [file mSystems.00077-20-sf004.tif]

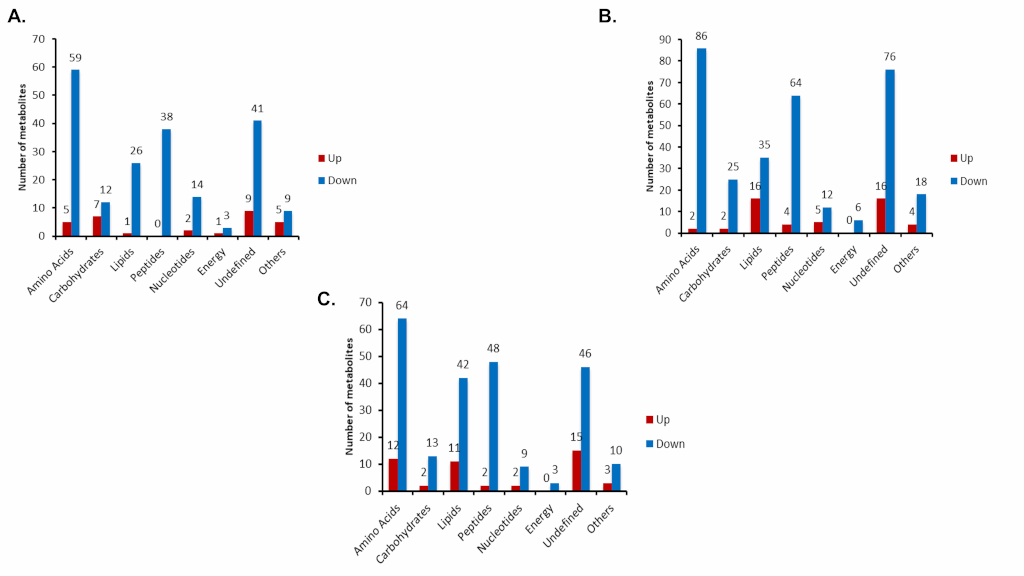

Supplement: FIG S5 [file mSystems.00077-20-sf005.tif]

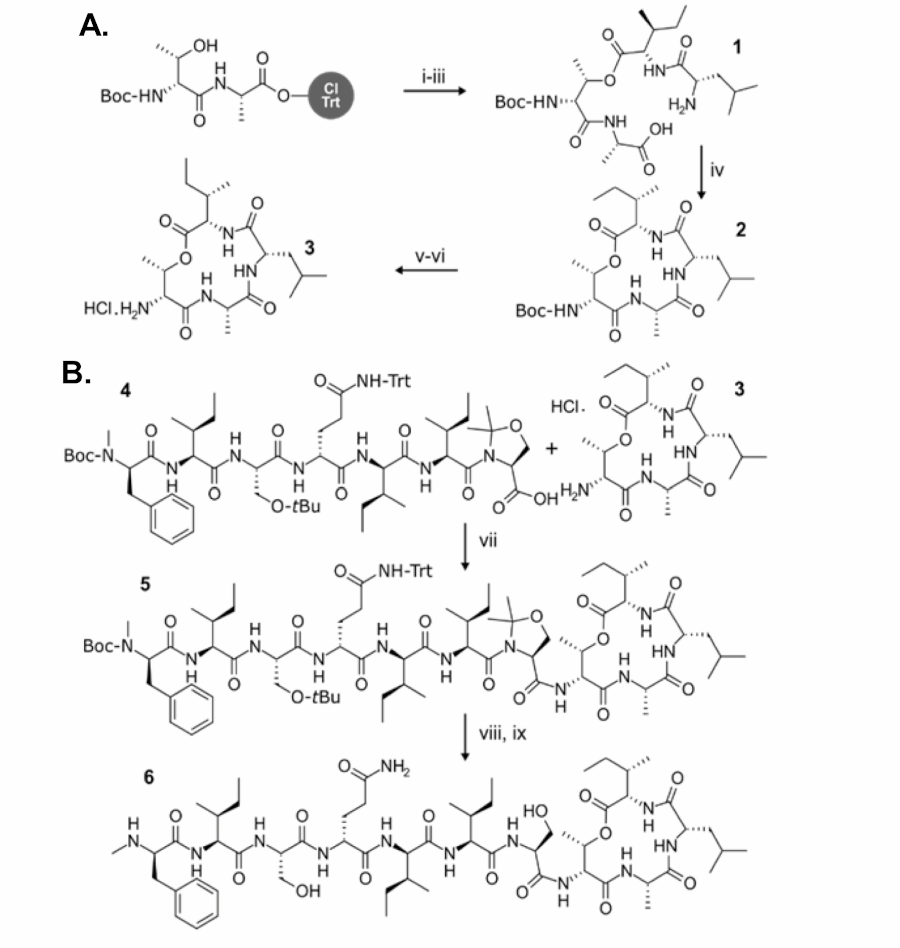

Supplement: FIG S6 [file mSystems.00077-20-sf006.tif]
