# Supplementary material for: The Killing Mechanism of Teixobactin against Methicillin-Resistant Staphylococcus aureus: an Untargeted Metabolomics Study
Source: mSystems. 2020 May 26;5(3):e00077-20. doi: 10.1128/mSystems.00077-20 (PMC7253363; doi:10.1128/mSystems.00077-20)
Supplement: TABLE S1 [file mSystems.00077-20-st001.pdf]

	Median RSD %		
	**1h**	**3h**	**6h**
**Control (Untreated)**	27	18	22
**Teixobactin**	20	23	25
